# Sparsity-based super-resolved coherent diffraction imaging of one-dimensional objects

**DOI:** 10.1038/ncomms9209

**Published:** 2015-09-08

**Authors:** Pavel Sidorenko, Ofer Kfir, Yoav Shechtman, Avner Fleischer, Yonina C. Eldar, Mordechai Segev, Oren Cohen

**Affiliations:** 1 Department of Physics and Solid State Institute, Technion, Haifa 32000, Israel; 2 Department of Chemistry, Stanford University, Stanford, California 94305, USA; 3 Department of Physics and Optical Engineering, Ort Braude College, Karmiel 21982, Israel; 4 Department of Electrical Engineering, Technion, Haifa 32000, Israel

## Abstract

Phase-retrieval problems of one-dimensional (1D) signals are known to suffer from ambiguity that hampers their recovery from measurements of their Fourier magnitude, even when their support (a region that confines the signal) is known. Here we demonstrate sparsity-based coherent diffraction imaging of 1D objects using extreme-ultraviolet radiation produced from high harmonic generation. Using sparsity as prior information removes the ambiguity in many cases and enhances the resolution beyond the physical limit of the microscope. Our approach may be used in a variety of problems, such as diagnostics of defects in microelectronic chips. Importantly, this is the first demonstration of sparsity-based 1D phase retrieval from actual experiments, hence it paves the way for greatly improving the performance of Fourier-based measurement systems where 1D signals are inherent, such as diagnostics of ultrashort laser pulses, deciphering the complex time-dependent response functions (for example, time-dependent permittivity and permeability) from spectral measurements and *vice versa*.

## Abstract (web summary)

In measurements that employ phase retrieval algorithms, such as coherent diffraction imaging, reconstruction of one-dimensional signals is challenging due to ambiguity issues. Here, the authors demonstrate super-resolution coherent imaging of one-dimensional objects by utilizing sparsity prior information.

## Graphical abstract

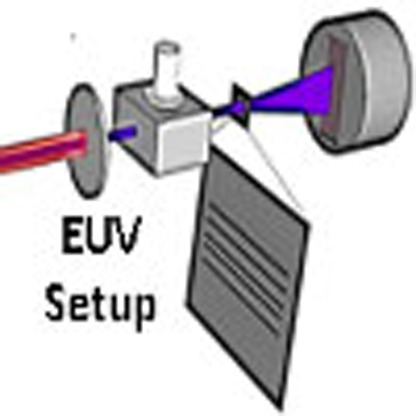

Phase-retrieval algorithms, aimed at reconstructing signals from the magnitude of their Fourier transform, are used in many fields of science and engineering^1^,^2^,^3^,^4^,^5^,^6^,^7^,^8^,^9^,^10^,^11^,^12^,^13^, including radar^7^, astrophysics^8^, nuclear magnetic resonance^9^, electron microscopy^10^, diagnostics of short pulses^11^,^12^ and spectroscopy^13^. A prime example and an important application for phase retrieval is coherent diffraction imaging (CDI)^1^,^2^,^3^,^4^,^5^,^6^ where an object is algorithmically reconstructed from measurements of the freely diffracting intensity pattern image that corresponds to the spatial spectrum (that is, the square of the Fourier amplitude) of the object. Generally, measuring only the Fourier magnitude lacks significant portion of the information and leads to an underdetermined system of equations. To try and compensate for this, additional information is necessary. A standard approach since 1982 is to use the advance knowledge about the support of the imaged object, that is, knowing the boundaries within which the object is confined^14^. Over the years, CDI evolved into an important lens-less imaging method, which is especially attractive for microscopy with coherent extreme-ultraviolet and x-ray radiation^1^,^2^,^3^,^4^,^5^,^6^ because optical components (lenses and mirrors) in these spectral regions are much less available than in the visible spectral region.

A key issue in CDI, and more generally in all phase-retrieval methods, is the uniqueness (or ambiguity) of the solutions^15^. Namely, is there only one object that corresponds to the measured spectrum and the prior information at hand (up to trivial ambiguities including global phase shift, conjugate inversion and spatial shift)? How is uniqueness affected by noise, which is always present in measurements? CDI had become a popular technique because uniqueness can often be achieved using prior knowledge about the support of two-dimensional (2D) and 3D objects^1^,^2^,^3^,^4^,^5^,^6^,^14^. Even so, finding this unique solution is often challenging because there is no general and robust method guaranteed to find the solution in a stable manner. In sharp contradistinction with 2D and 3D objects, the situation with 1D objects is far worse: constraints on the support of the sought signal are known not to guarantee uniqueness^16^,^17^,^18^. It was also shown that reconstruction using maximum entropy algorithms fails to remove the ambiguity^19^ and direct phase-retrieval approaches are highly sensitive to noise^20^. Thus, instead of strictly-algorithmic methods, hardware-based approaches (multiple measurements) have been used for reliable recovery of 1D signals from their Fourier magnitude measurements^11^,^21^,^22^,^23^. Clearly, hardware-based methods increase the complexity of the measurement apparatus. For example, Frequency Resolved Optical Gating (FROG), a popular technique for diagnostics of ultrashort laser pulses, transforms the 1D phase-retrieval problem into a 2D problem^11^. In another example, a 1D (line) x-ray field was retrieved from multiple far-field intensity patterns obtained by moving an aperture at the beam's focus^24^. Other examples include vectorial phase retrieval^22^ and CDI with multiple structured illumination patterns^23^, both relying on transforming the original 1D problem into a 2D counterpart. Distinct from these hardware approaches, it was recently shown (in theory and simulations) that using a sparsity prior (that is, using the prior information that the sought signal can be represented in a compact form in a known mathematical basis) can give rise to uniqueness in phase retrieval of 1D signals under certain conditions^25^. This article presents the first experimental demonstration of sparsity-based phase retrieval of sparse 1D signals. Specifically, we demonstrate sparsity-based 1D CDI. Moreover, we show that employing the sparsity prior often enables super-resolution, recovering the 1D object beyond the physical resolution limit of the imaging system.

Essentially, the sparsity prior corresponds to having advance knowledge that the unknown signal has some characteristic structure. The simplest case occurs when the ‘sparsity basis' (a mathematical basis in which the object is represented compactly) is known in advance. But even more generally, the sparsity basis can be extracted (learned), under certain conditions, from the measurements themselves or from data with similar features that is often available from other sources^26^. Using sparsity as a ‘prior' is very powerful because, on one hand it is general (it does not limit the signal to a specific form), and on the other hand it can remove ambiguities. The sparsity prior has been used extensively in many fields of engineering (for example, data and image compression), statistics and mathematics^27^, and it leads to robust recovery even in the presence of significant noise^27^,^28^,^29^,^30^. In the context of optics, sparsity has been employed in various applications ranging from single pixel camera^31^, compressive holography^32^, compressive ghost imaging^33^, diagnostics of coherent modes^34^ and un-mixing using spectral measurements^35^ to super-resolution and sub-wavelength imaging^36^. It was also proposed^37^ and demonstrated in simulations^38^ that the use of sparsity can enhance Ankylography, and make it possible to recover the 3D structure of complex molecules. Importantly, recent work has demonstrated sparsity-based super-resolution in phase retrieval of 2D objects by CDI^29^,^39^. The resolution of CDI is set by the highest measured spatial frequency, which is determined by the ratio between the size of the detector array (typically a CCD camera) and the distance from object to measurement plane, and by the signal-to-noise ratio. However, beyond such signal-to-noise and the geometrical issues, the free-space transfer-function of electromagnetic waves is essentially a low-pass filter with a cutoff at 1/*λ*, leading to the well-known ‘diffraction limit' (that is, the fundamental limit on imaging resolution is ∼*λ*/2, *λ* being the wavelength of the light). Our recent work on CDI has demonstrated, theoretically and experimentally, that employing sparsity can facilitate enhanced resolution even far beyond the fundamental diffraction limit^29^. However, in that experiment we used visible radiation: a spectral region which is incompatible with the most important CDI applications, such as measuring the structures of bio-molecules using x-ray laser pulses and semiconductors mask metrology using extreme-ultraviolet (EUV) radiation.

Here we present sparsity-based super-resolved phase retrieval of 1D signals. We show that the sparsity prior can often remove the ambiguity associated with the loss of phase in 1D information and in parallel also yield super-resolution: the recovery of high spatial frequencies considerably beyond the measurement range in Fourier space. We demonstrate experimentally that sparsity can be utilized for super-resolution in CDI of 1D objects. Specifically, we demonstrate resolution enhancement up to ∼4.5 times beyond the inherent resolution limit of our CDI microscope. Notably, some of the CDI microscopy experiments presented here uses EUV radiation, thus our observation extends the concept of sparsity-based super-resolution imaging into the range of very short-wavelengths. Utilizing the sparsity prior to recover the structure of 1D information from the measurement of its Fourier magnitude also paves the way to sparsity-based phase retrieval in other applications, including complete diagnostics of ultrashort pulses, spectroscopy and more.

## Results

### Simulations

In CDI, the image is algorithmically reconstructed from the intensity diffraction pattern and some prior information about the object^1^,^2^,^3^,^4^,^5^,^6^,^40^. As we have shown recently, using sparsity as the prior can be very powerful in CDI^29^,^39^. Indeed, it was proposed in numerical simulations that sparsity can remove the ambiguity associated with 1D phase retrieval (but without super-resolution)^25^,^28^,^30^,^41^. Moreover, ref. 30, developed a new sparsity-based phase-retrieval algorithm termed GESPAR (greedy sparse phase retrieval), which is based on utilizing a fast local search method^42^ and optimization of a sparsity-constrained nonlinear objective function. Here we use GESPAR in CDI.

**Figure 1:**
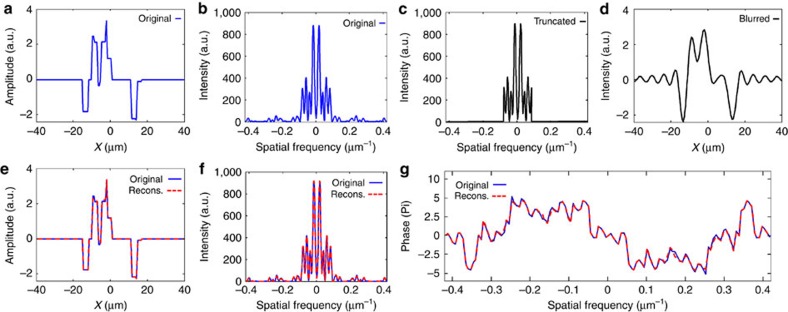
Numerical demonstration of super-resolved 1D CDI. (**a**) The ‘original' 1D object which consists of seven rectangular functions of 3-μm width, with different amplitudes and centres (some rectangles overlap). (**b**) Power spectrum of the original object. (**c**) Truncated power spectrum corresponding to the part used to simulate the measured data, with 40 dB noise added. (**d**) The blurred reconstruction calculated by inverse Fourier transform of the ‘measured' power spectrum presented in **c** assuming complete knowledge of the spectral phase. (**e**) Sparsity-based reconstruction (dashed red) compared with the original object (solid blue). The reconstruction uses the ‘measured' power spectrum (of **c**) and the prior information that the original object is sparse in the basis of shifted rectangular functions. Extrapolated power spectrum (**f**) and recovered spectral phase (**g**) calculated via sparsity-based reconstruction (dashed red) compared with the original object (solid blue).

We first present a simulated example of sparsity-based super-resolution 1D CDI. Figure 1a shows a signal (which we term the ‘original object') consisting of seven rectangles with 3-μm width and different amplitudes and centres (notice that some of the rectangles overlap). The amplitudes correspond to the optical transmission function through structured holes made in an opaque mask. When the mask in illuminated by a plane wave (or a broad collimated beam), the phase is uniform, hence the amplitudes have real values (up to some unimportant global phase). The power spectrum of the original object is shown in Fig. 1b. As shown in the Supplementary Note 1 and Supplementary Figs 1–2, the problem of reconstructing the original 1D object from the power spectrum suffers from ambiguity that cannot be removed by prior information about the support of the object. We demonstrate below that sparsity removes the ambiguity and at the same time facilitates super-resolution. To demonstrate super-resolution, we truncate the power spectrum and add 40 dB of white Gaussian noise to obtain the ‘truncated power spectrum' emulating a physical measurement in a typical CDI system (Fig. 1c). Reconstruction requires retrieval of the phase of the spatial spectral field. Figure 1d displays the object that corresponds to the truncated power spectrum of Fig. 1c, while assuming that the spectral phase is known (in this example, the correct phase is simply calculated by a Fourier transform of the original object (Fig. 1a)). Naturally, this reconstructed object (Fig. 1d) is a blurred version of the original object. That is, the incomplete power spectrum leads to considerable loss of resolution even if the spectral phase is known. Next, we implement sparsity-based reconstruction on the truncated spatial power spectrum, without assuming any knowledge on the spectral phase. As a model, we assume that the object is constructed from a small (unknown) number of the following basis functions: rectangles of 3-μm width, at positions that are limited to a particular grid of 1,024 points. The sparsity-based GESPAR reconstruction algorithm^30^ finds the number of rectangles, their locations and their amplitudes from the truncated power spectrum (Fig. 1c). The reconstructed object is shown in Fig. 1e by the dashed red curve. Its power spectrum and spectral phase are displayed in Fig. 1f,g, respectively (dashed red). To enable comparison, Fig. 1e,f also shows the original object (solid blue curves). Clearly, the reconstructed object, its complete power spectrum and its reconstructed spectral phase match the original object very well, despite the fact that our ‘measured data' is the noisy, truncated, power spectrum that also lacks any knowledge on the spectral phase.

**Figure 2:**
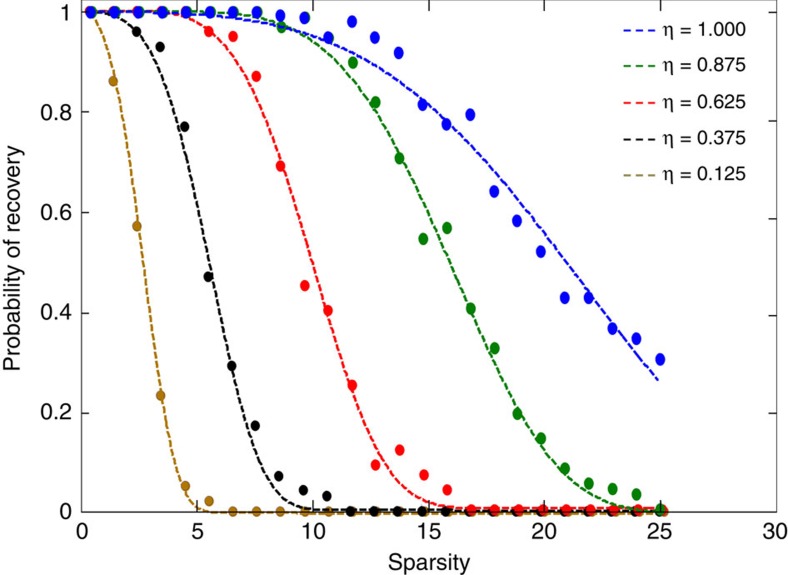
Calculated probability for correct CDI reconstruction as a function of the sparsity level of the 1D signal. Here *η* is defined as the ratio between the maximal ‘measured' frequency and the highest frequency of the sampled signal, 1.28 μm^−1^.

Figure 1 demonstrates that sparsity-based super-resolution 1D CDI is possible. However, it is important to evaluate its performance. To do that, we test our method on many objects comprised of random distributions of the rectangles and varying levels of truncation of the power spectrum (the truncation level, *η*, is defined by the ratio between the maximal ‘measured' frequency and the highest frequency of the sampled signal, 1.28 μm^−1^). Figure 2 shows the probability of successful recovery versus sparsity level, *s* (number of rectangles), in the original object, for different truncation levels. Each point in this graph is obtained by running our reconstruction algorithm over 100 random signals on a grid of 128 points. We define the reconstruction as successful if the relative error (defined as 
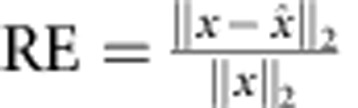
, where *x* and 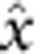 are the original and recovered signals, respectively), between the original and reconstructed objects is <0.01. It is important to note that the upper sparsity limit for successful reconstruction can be increased by increasing the oversampling^30^. This plot clearly shows that the sparsity prior can remove the ambiguity (inherent to 1D phase-retrieval problems) even if only part of the spectrum is detected. Moreover, the plot shows that there is a large range of parameters (sparsity and truncation level) within which GESPAR reconstruction is reliable.

### Experimental example with a binary object

**Figure 3:**
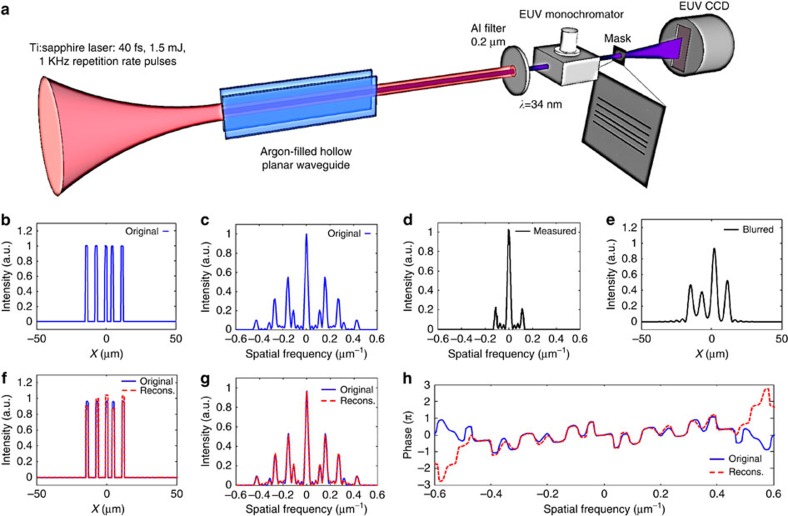
Experimental demonstration of super-resolution CDI of an effectively one-dimensional object. (**a**) Experimental Set-up. The imaged object consists of five stripes, each of 2-μm width, as shown by the scanning electron microscope (SEM) image zoomed-in expanded from the mask. (**b**) The real-space 1D object and (**c**) its spatial power spectrum, that conform to the mask SEM image (shown in **a**) playing the role of the ‘original' object. (**d**) Measured intensity pattern which approximately corresponds to a truncated power spectrum of **c**. (**e**) The blurred object calculated by inverse Fourier transform of the product: square root of the measured intensity times the correct phase. (**f**) Sparsity-based reconstruction (dashed red) compared with the original object (solid blue). (**g**) Recovered power spectrum and (**h**) spectral phase calculated through sparsity-based reconstruction (dash red) compared with these functions calculated by a Fourier transform of the original object.

Next, we demonstrate the concept of sparsity-based super-resolution in 1D CDI in imaging experiments. The experimental set-up is shown in Fig. 3a. The EUV light is generated using high harmonic generation. A train of 40-fs-long pulses with central wavelength of 0.8 μm and 1.5 mJ per pulse emerging from a Ti:Sapphire laser amplifier system at rep rate of 1 kHz is focused into a hollow planar waveguide (200 μm inner diameter) filled with argon at 22 torr. In this regime, the gas emits a comb of coherent high-order odd harmonics that can be used for CDI^43^. A 200-nm-thick Al film blocks the infrared driving laser. An EUV monochromator selects a single harmonic-order at *λ*∼35 nm. The imaged object is a 200-nm-thick Zirconium mask, where 5 bars of 2 μm were etched out (see scanning electron microscope image in Fig. 3a). Zirconium is opaque for this wavelength so the EUV light is transmitted only through the bars. Hence, the optical image is made up of the EUV light transmitted through this mask, which consists of these five stripes. Our EUV source is spatially coherent and located about 2.5 m before the mask, therefore the assumption of plane wave illumination is valid. The far-field diffracted light is recoded (25-min exposure time) by an x-ray CCD camera (1,024 × 256 pixels) placed 72 cm after the mask. The sought information in our object is practically 1D; hence we integrate the detected intensity pattern along the uniform (horizontal) dimension (256 pixels). Figure 3b shows the one-dimensional real-space information calculated by integrating the scanning electron microscope (SEM) image along the horizontal axis. Thus, in our experiment, the plot in Fig. 3b corresponds to the original object. The calculated absolute value squared of the Fourier transform of the 1D object is shown in Fig. 3c. Physically, this represents the spatial power spectrum of the 1D object of Fig. 3b. In the experiment, however, we measure only a fraction of the entire power spectrum, as the far-field diffraction pattern of the mask is limited by the dimensions of our CCD camera. The measured truncated spatial power spectrum is shown in Fig. 3d. Comparing Fig. 3d with Fig. 3c reveals that a significant part of the spatial power spectrum is lost. Namely, not only do we detect only the far-field intensity (while completely missing the Fourier phase information), but in addition our measured power spectrum is severely truncated. Expectedly, the object corresponding to the detected (highly truncated) power spectrum is considerably blurred, even if we assume we do know the correct spectral phase (Fig. 3e). Indeed, the theoretical resolution of this image, according to the Nyquist–Shannon sampling theorem^44^, is ∼10 μm, while in reality the features (the bars) are five times narrower: 2-μm wide. Thus, to reconstruct the original object from the detected spatial spectrum, we need to retrieve both the lost part of the spectrum and the phase distribution over the entire spectral span. Our algorithmic reconstruction, using GESPAR, is displayed in Fig. 3f,g. Here we assume that the image consists of a small number of 2-μm-wide rectangles that are spanned on a grid with 1,024 points. Clearly, we are able to recover the correct number of bars, their positions, their amplitudes, the missing part of the spectrum and the correct phase in both the measured and the absent parts of the spectrum (the original and reconstructed phase differ significantly only in the region in which the power spectrum diminishes). Consequently, while we measured the power spectrum of the object up to spatial frequency 0.13 μm^−1^, we reconstructed its spatial spectral amplitude and phase with good fidelity up to 0.46 μm^−1^. That is, we increased the bandwidth and resolution by 3.5 times. This experiment shows that our sparsity-based super-resolved phase-retrieval reconstruction is robust and can be implemented under experimental conditions typical in optical CDI settings. A second experimental demonstration of super-resolution in our EUV CDI microscope, in this case of a symmetric object consisting of 7 bars, is shown in the Supplementary Note 2 and Supplementary Fig. 3.

**Figure 4:**
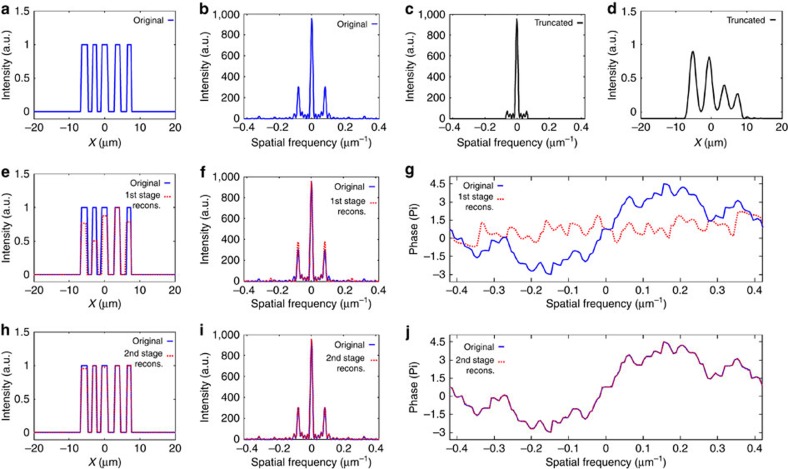
Numerical demonstration of sparsity-based super-resolved 1D CDI of objects consisting of rectangles with only approximately known widths (allowing 20% variations). (**a**) The ‘original' 1D object which consists of five rectangular functions with widths (left to right) 2.4, 1.6, 2.4, 2 and 1.6 μm. (**b**) Power spectrum of the original object. (**c**) Truncated power spectrum used to simulate the measured data, with 35 dB noise added. (**d**) The blurred reconstruction calculated by inverse Fourier transform of the ‘measured' power spectrum presented in **c** assuming complete knowledge of the spectral phase. (**e**–**g**) Sparsity-based reconstruction (dashed red) compared with the original object (solid blue). The reconstruction uses the ‘measured' power spectrum (of **c**) and performed with GESPAR in the basis of shifted rectangles with a fixed width of 2 μm. (**h**–**j**) Sparsity-based reconstruction (dashed red) compared with the original object (solid blue). The reconstruction uses the same ‘measured' power spectrum (of **c**) but performed with a modified GESPAR algorithm implemented in a basis of shifted bars with variable widths (see Supplementary Note 3).

Figure 3 presents an example of sparsity-based super-resolution 1D CDI of an object that is sparse in a basis of rectangles with a known fixed width. One may therefore question what happens if the widths of the rectangles are known only approximately. Such a problem can arise in searching and characterizing defects in microelectronic chips. To this end, we introduce a second stage in our reconstruction algorithm. Namely, the first stage corresponds to the algorithm used in Figs 1, 2, 3, which assumes rectangles with constant widths, while the second stage is more general: it finds the deviations in the widths of the rectangles from the initially assumed width. For a detailed description of the second-stage algorithm see the Supplementary Note 3. Using simulations, we verify that our algorithm works very well when the uncertainty in the width of the rectangles (the bars in Fig. 3) is up to 20%. A numerical example is presented in Fig. 4. The ‘original object' (Fig. 4a) is based on the object in Fig. 3, but this time we introduced deviations in the widths of the bars. The widths in this specific example are, from left to right in Fig, 4a—2.4, 1.6, 2.4, 2 and 1.6 μm. The full power spectrum of the original object is shown in Fig. 4b. Figure 4c displays the truncated power spectrum with added 35 dB of white Gaussian noise, emulating the CDI measurement. Figure 4d displays the object that corresponds to the truncated power spectrum of Fig. 4c (that is, a blurred version of the original object), while assuming that the correct spectral phase is known. In this example, since the widths of the bars are not the same, we expect that the first stage algorithmic reconstruction would not be able to recover the information accurately. Indeed, applying our first-stage reconstruction algorithm (which assumes that the object consists of a small (unknown) number of rectangles of constant known width of 2 μm), yields the incomplete recovery presented in Fig. 4e–g. More specifically, the first-stage reconstruction finds the correct number of rectangles and their approximate positions, but their amplitudes and, of course, their widths are clearly wrong. However, when we apply the second-stage algorithm we obtain the excellent reconstruction show in Fig. 4h–j. The tiny residual errors in the amplitudes (in Fig. 4h) are due to the noise we added to the truncated power spectrum.

### Experimental example with a smooth signal

**Figure 5:**
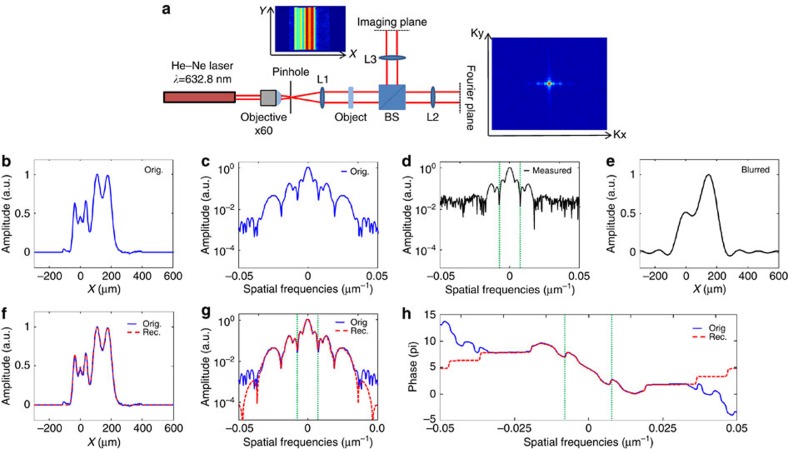
Experimental demonstration of sparsity-based super-resolved 1D CDI of a continuous image. (**a**) Experimental set-up. He–Ne laser is spatially filtered and collimated by the objective, pinhole and lens L1. The spatially coherent beam illuminates a transmission mask. The spatial power spectrum of the light going through the mask is measured by a CCD camera positioned at the focal plane of the lens L2 with 100 mm focal length. The mask is also imaged directly using a lens with L3=200 mm focal length. (**b**) 1D object obtained by integrating the real-space image along the uniform direction, which plays the role of the ‘original' image. (**c**) The power spectrum of the original image. (**d**) The measured power spectrum. The vertical dashed lines mark a stepwise low-pass filter (LPF) at 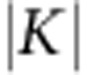
≤0.0083 μm^−1^. The ‘measured low-resolution power spectrum' corresponds to the measured filtered power spectrum with the LPF. (**e**) The blurred reconstruction calculated by inverse Fourier transform of ‘measured low-resolution power spectrum', assuming complete knowledge of the spectral phase. (**f**) Sparsity-based reconstruction (dashed red) compared with the original object (solid blue). The reconstruction uses the ‘measured low-resolution power spectrum' and the prior information that the original object is sparse in the basis of shifted Gaussian functions. Extrapolated power spectrum (**g**) and recovered spectral phase (**h**) calculated via sparsity-based reconstruction (dashed red) compared with the original object (solid blue).

Figures 1, 2, 3, 4 present examples of sparsity-based super-resolution 1D CDI of objects that are sparse in a basis of rectangles. Another example, presented in the Supplementary Note 4 and Supplementary Fig 4, extends the rectangular basis to a frame that includes rectangles with several different widths. In principle, our sparsity-based method works as well with triangles or other choices of localized waveforms (as demonstrated in the Supplementary Information of ref. 30). The rectangle basis is appropriate for piecewise-constant objects, but it is not an imperative part of our method. In fact, our methodology can be used to reconstruct a large variety of objects, given that the objects have structure and hence they can be represented compactly in some (rather general) mathematical basis. This point is highlighted in the next experiment, which demonstrates super-resolved 1D CDI of a continuous object. Here we use a frame of shifted Gaussians with different widths: the basis functions are 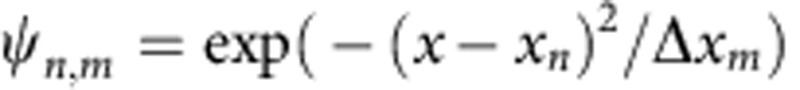, where *x*_*n*_ and Δ*x*_*m*_ are centres and widths of Gaussians, respectively. This frame is useful for compact representation of smooth objects with exponential decay. In the current example, we use *x*_*n*_=−100+20*n* for *n*=1, 2, 3, … 20 and Δ*x*_*m*_=5+5*m* for *m*=1, 2, 3, … 10 hence our frame consisted of 200 functions. In this experiment, we use a CDI microscope that uses light from a He–Ne laser (*λ*=632.8 nm). In addition to the power spectrum, the microscope also records the real-space image of the object (a partially transparent film). The experimental set-up and the recorded real-space image and spatial power spectrum of the object are shown in Fig. 5a. The sought information in our object is 1D; hence we integrate the detected intensity patterns along the uniform (vertical) dimension in the object and Fourier planes (that is, along y and ky in Fig. 5a). The resulted integration of the real-space image is shown in Fig. 5b. In our experiment it plays the role of the original object. The calculated absolute value squared of the Fourier transform of the original object (which represents the calculated spatial power spectrum of the original object) is shown in Fig. 5c. The measured spatial power spectrum is shown in Fig. 5d. As shown, the measured power spectrum at very high spatial frequencies is dominated by noise. Thus, the bandwidth of the measured power spectrum is practically truncated by the noise. To highlight the strength of our technique, we further truncate the measured power spectrum, by numerically applying a step function low-pass filter with a cutoff spatial frequency at 0.0083 μm^−1^ (green dashed lines in Fig. 5d). Figure 5e displays the blurred object that corresponds to the truncated power spectrum of Fig. 5d, while assuming that the spectral phase is known (in this example, the correct phase is simply calculated by a Fourier transform of the original object (Fig. 5b)). Next, we apply our sparsity-based reconstruction algorithm (GESPAR) which searches for an object that is represented most compactly in the Gaussian frame among all the objects conforming to the measured power spectrum (after the additional numerical truncation). Our algorithm finds such an object that is (most compactly) represented by seven functions in the Gaussian frame. The reconstructed object, along with its reconstructed power spectrum and spectral phase are shown in Fig. 5f–h. These results clearly show that sparsity-based reconstruction works very well despite the fact that the object is a 1D smooth function and that the measured data is noisy, truncated and lacks any knowledge on the spectral phase. The spectra of the reconstructed and original objects coincide within a bandwidth of ±0.037 μm^−1^, which corresponds to 4.5 times the bandwidth of the low pass filter. In other words, Fig. 5 presents × 4.5 super-resolved 1D CDI.

### Summary and outlook

We presented numerical and experimental phase retrieval of 1D signals combined with bandwidth extrapolation (super-resolution), by employing prior knowledge that the sought information is sparse in a known basis or in a mathematical frame. More generally, the sparsity basis should be selected, or potentially learned, from the actual measured data or from other available sources, according to the type of objects that are imaged. Thus, we believe that sparsity-based CDI (for 1D, 2D and 3D objects) can be very general, as most imaged objects can be represented compactly in an appropriate basis. In fact, this is also the logic behind many popular image compression techniques, such as JPEG^45^. This work paves the way to significant progress in many other ill-posed problems where 1D signals are inherent, such as deciphering the complex time-dependent response functions of materials and structures from spectral measurements and *vice versa*.

## Additional information

**How to cite this article:** Sidorenko P. *et al*. Sparsity-based super-resolved coherent diffraction imaging of one-dimensional objects.. *Nat. Commun.* 6:8209 doi: 10.1038/ncomms9209 (2015).

## Supplementary Material

Supplementary Information Supplementary Figures 1-4, Supplementary Notes 1-4 and Supplementary References
