# Supplementary material for: Sparsity-based super-resolved coherent diffraction imaging of one-dimensional objects
Source: Nat Commun. 2015 Sep 8;6:8209. doi: 10.1038/ncomms9209 (PMC4569841; doi:10.1038/ncomms9209)
Supplement: Supplementary Information — Supplementary Figures 1-4, Supplementary Notes 1-4 and Supplementary References [file ncomms9209-s1.pdf]

Supplementary Figures

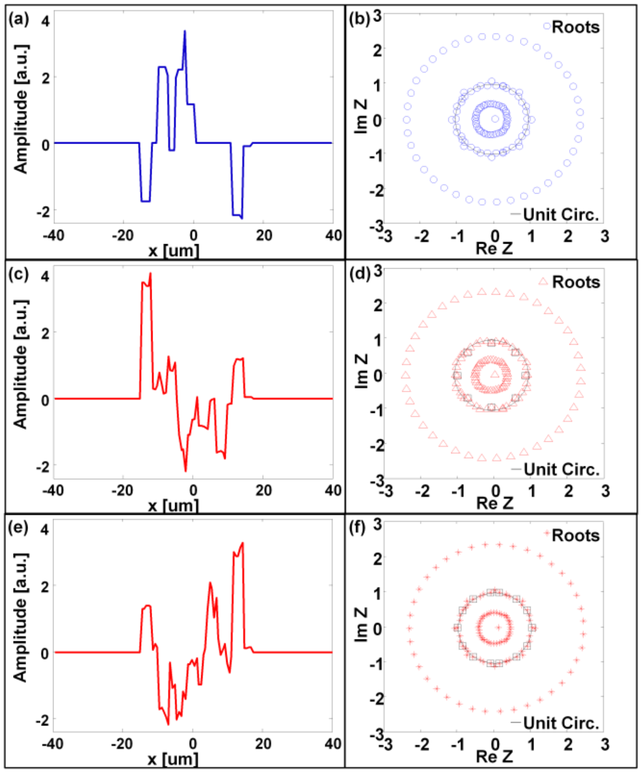

Supplementary Figure 1: Demonstration of the ambiguity involved in reconstructing the 1D object presented in Fig. 1 in the paper. (a) Original object which consists of 7 stripes of 3μm width. The object is sampled on a 128 point grid. (b) Roots of the complex polynomial (of degree 127) that coincides with the discrete Fourier transform (DFT) of the original signal. (c) A signal with the same Fourier magnitude as that of (a). This signal is represented in the rectangle basis by no less than 43 elements. (d) The corresponding DFT roots (marked by red asterisks) of the signal in (c). This signal is obtained by transforming some of the original roots via $z \rightarrow 1/\bar{z}$; transformed roots are marked with black quadrangles. It is well known that exchanging roots by their conjugate reciprocals does not affect the magnitude of the spectrum. (e) Another signal with the same Fourier magnitude. This signal is represented in the rectangle basis by no less than 92 elements, and is obtained by a different set of roots transformed by their conjugate reciprocal (f).

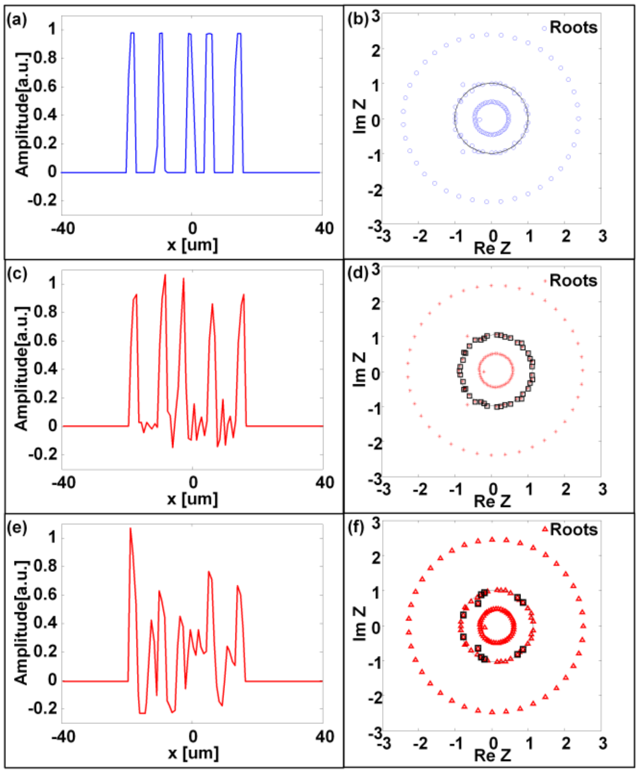

Supplementary Figure 2: Demonstration of the ambiguity involved in reconstructing the 1D object presented in Fig. 3 in the paper. (a) The original object which consists of 5 stripes. The object is sampled on a 128 point grid. (b) Roots of the complex polynomial (of degree 127) that coincides with the discrete Fourier transform (DFT) of the original signal. (c) A signal with the same Fourier magnitude as that of (a). This signal is represented in the rectangles basis by no less than 79 elements, and (d) its corresponding DFT roots (marked by red asterisks). This signal is obtained by transforming some of the original roots via→ $1/\bar{z}$; transformed roots are marked with black quadrangles; exchanging roots by their conjugate reciprocals does not affect the magnitude of the spectrum. (e) Another signal with the same Fourier magnitude. This signal is represented in the rectangles basis by no less than 103 elements, and is obtained by a different set of roots transformed by their conjugate reciprocal (f).

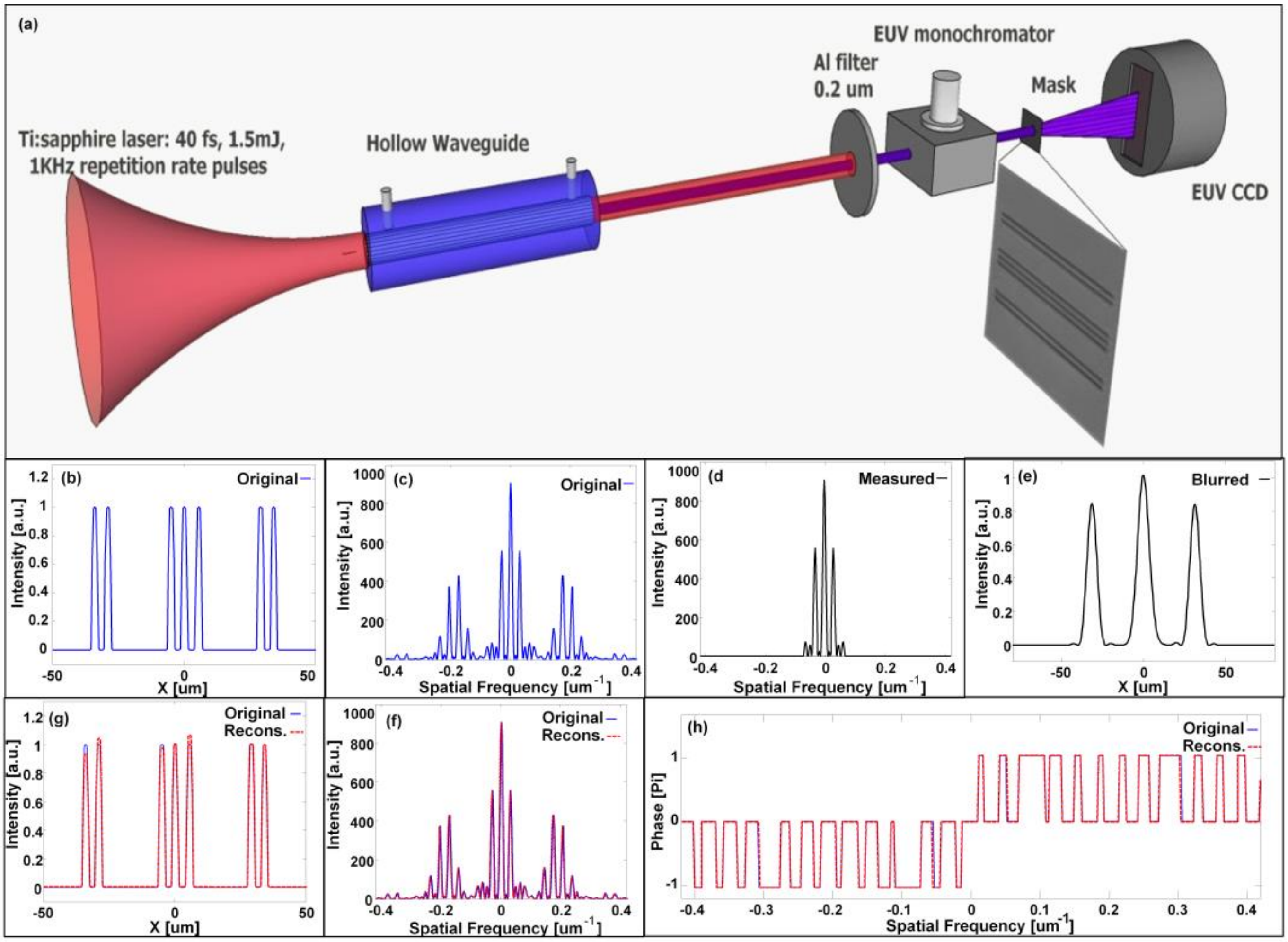

Supplementary Figure 3: Experimental demonstration of super-resolution CDI of a symmetric effectively one-dimensional object. (a) Experimental Setup. Real-space 1D image (b) and spatial power spectrum (c) that conform to the mask SEM image (shown in panel (a)), playing the role of the "original" image. (d) Measured intensity pattern with 10 minutes exposure (after integration over the non-diffraction dimension and scaled to spatial frequencies). (e) A blurred real-space amplitude that is calculated by inverse Fourier transform of the square root of the measured intensity and phase function that is calculated from the SEM image. (g) Sparsity-based reconstruction (dashed red) compared with the original image (solid blue). The retrieved power spectrum (f) and spectral phase (h) through the sparsity-based reconstruction (dash red) compared with these functions calculated by Fourier transform of the original image. Applying our method for efficient phase retrieval of sparse signals (our prior knowledge is that the sought information is sparse in the basis of shifted rectangles) leads to ~6 times enhanced resolution beyond the inherent resolution limit of our CDI microscope.

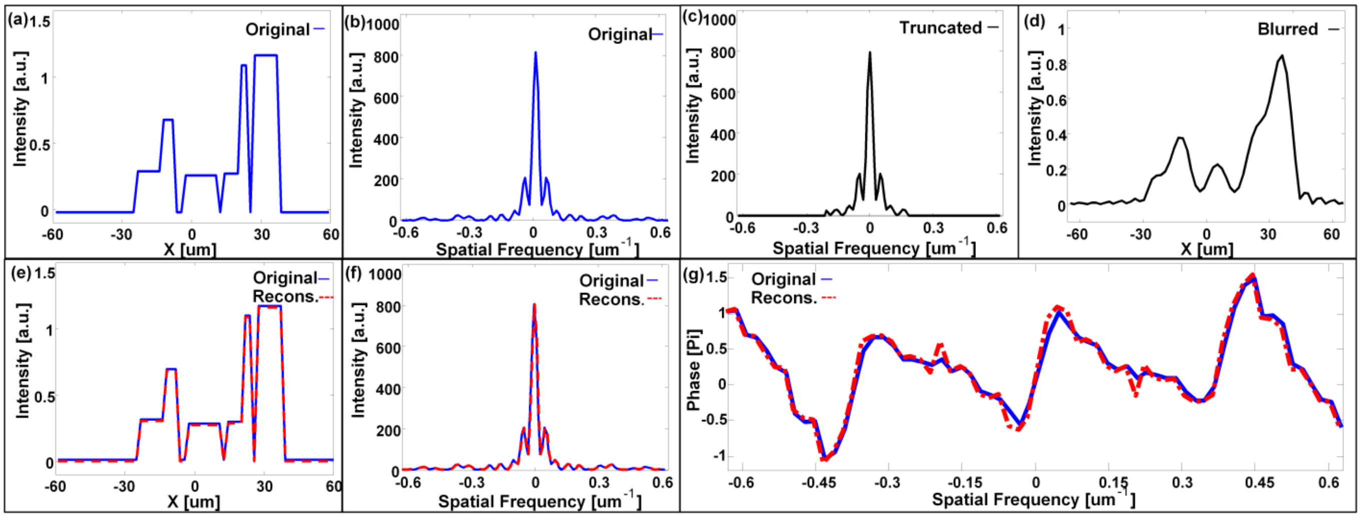

Supplementary Figure 4: Super-resolved CDI of a 1D dense piecewise-constant object. (a) The "original" 1D object. (b) Power spectrum of the original object with 43dB noise. (c) Truncated power spectrum that corresponds to the part used to simulate the measured data. (d) The blurred reconstruction calculated by inverse Fourier transform of the "measured" power spectrum presented in (c), assuming full knowledge of the spectral phase. (e) Sparsity-based reconstruction (dashed red) compared with the original image (solid blue). The reconstruction uses the "measured" power spectrum (of (c)) and the prior information that the original image is sparse in the frame of shifted rectangular functions with different widths. Extrapolated power spectrum (f) and recovered spectral phase (g) calculated via sparsity-based reconstruction (dashed red) compared with the original image (solid blue).

Supplementary Note 1

Demonstration of ambiguity in 1D CDI of example objects in the paper

It is well known that 1D CDI is generally an ill-posed problem: Generic compact support objects correspond to the same far-field intensity pattern[2-3]. Still, there are some uncommon objects for which 1D CDI can yield unique solutions where knowing the support is the only prior information. In this section, we demonstrate that the theoretical (Fig. 1) and experimental (Fig. 3) examples in the paper do not belong to those unusual cases, but rather belong to the general class of signals that do suffer from the ambiguity problem characteristic to the problem of phase retrieval of 1D objects.

We begin with the object in Fig. 1 which is also plotted in Supplementary Figure 1 a. It consists of 7 rectangle functions with 3 μm width with different amplitudes and centers (some rectangles overlap). Figure S.1b shows the roots (zeros) of the complex polynomial function that corresponds to the electric field spectrum[3]. If all the roots were on the unit circle (i.e. their absolute values are 1), then the reconstruction of the object from the power spectrum and support prior was unique[3]. If all the roots were inside or outside of the unit circle - then the ambiguities were trivial[3]. However, this is not the case: as clearly shown in Supplementary Figure 1 b, the zeros are complex, are not on the unit circle and they are not limited to inside or outside of the unit circle. This proves that 1D phase retrieval of this object suffers from non-trivial ambiguities that cannot be removed by support prior information. The ambiguous signals can be readily demonstrated by flipping some of the zeros with respect to the unit circle[3]. As an example, consider Supplementary Figure 1 c – which shows such a signal that results

from flipping 41 zeros (the zeros are now shown in Supplementary Figure 1 d). Figure S.1e shows another signal, that results from flipping 12 zeros (the zeros are now shown in Supplementary Figure 1 f). Notably, all these signals (arising through zero flipping) have the same power spectrum, and can be represented as a superposition of rectangles with 3 μm width (which is a complete basis). But, only the original object is sparse (only 7 basis functions are required to describe it). The minimal number of base functions required for representing the erroneous reconstructions are significantly larger (73 and 91 for the signals in Supplementary Figures 1c and 1e, respectively). This shows that it is sparsity that distinguishes between the correct signal and the ambiguous signals. Supplementary Figure 2 shows the same result for the experimental object presented in Fig. 3 in the paper.

Supplementary Note 2

Experimental demonstration of super-resolved CDI of symmetric, effectively one dimensional, objects.

The following figure (Supplementary Figure 3) presents an example equivalent to Fig. 3 in the paper, with the exception that here the object (the sought information) is a symmetric image. The experimental setup is presented in Supplementary Figure 3 a. Pulses with 1.5 mJ energy/pulse and 40 fs pulse duration from a Ti:Sapphire laser amplifier system are focused into a fused silica 5 cm long hollow-fiber with 150 μm inner diameter that is filled with argon at 22 Torr. A 200 nm thick Al film blocks the driving laser. A EUV monochromator selects a single harmonic-order at λ~35 nm. The imaged object consists of a 200 nm zirconium film with 7 hollow stripes with 2 μm width (see SEM image in Supplementary Figure 3 a). The diffracted light is recoded (40 minutes exposure time) by an x-ray CCD camera (1024×256 pixels). The sought information in our object is practically 1D; hence we integrate the detected intensity pattern along the non-diffraction (horizontal) dimension (256 pixels). Examining the performance of our sparsity-based algorithm (utilizing prior knowledge that the sought information is sparse in the basis of shifted rectangles) by comparing the reconstructed information with the original image (Supplementary Figure 3 (g,f,h)) leads to ~6 times resolution enhancement (while we measured the power spectrum of the object up to spatial frequency $0.073um^{-1}$, we reconstructed its spatial spectral amplitude and phase with good fidelity up to $0.4um^{-1}$). The experimental object in this example is symmetric; a fact that in principle could assist the phase retrieval, yet symmetry was not used in our reconstruction.

Supplementary Note 3

Sparsity-based algorithm for super-resolved 1D CDI of objects that consist of rectangles with only approximately known widths.

Our problem of super-resolved phase retrieval can be written mathematically as

$$min_z \sum_{i=1}^{N}(|F_i(Dz)|^2 - y_i)^2 \quad (1)$$

$$s.t. ||z||_0 \leq s$$

Here $y \in R^N$ is the magnitude-squared of a N point DFT of a vector $x \in R^M$ with $N < M$, F_i is the ith-row of a $N \times M$ DTF matrix, $D \in R^{M \times K}$ is a dictionary where $x = Dz$ and $||.||_0$ stands for the zero-"norm" that counts the number of non-zero values in $z \in R^K$. In other words, our measurements vector y is a truncated power spectrum of the signal x, which can be represented sparsely in the basis D. In the simple case, D is known in advance (e.g. shift rectangles with constant width) and one can implement the GESPAR algorithm to solve the problem defined by Eq. (1) (as described in Ref 1 and as we implement in the reconstructions presented in Figs 1-3 in the paper). Below we describe the algorithm for super-resolved 1D CDI when the widths of the bars are known only approximately. Specifically, in the example presented in Fig. 4 in the paper we assume that the objects consist of rectangles of 2±0.4 um with step size of 0.1 um.

<u>Algorithm for basis update.</u>

Input: Measurements y, rectangle width value and uncertainty $w \mp \Delta$ with step δ, threshold parameter τ, maximal number of iterations $MaxIter$ and k.

Output: Estimate $\hat{z}$ of z

Initialize: Construct dictionary D consisting of shifted bars with constant width w. Set $k = 0$, solve problem (1) with GESPAR implementation and obtain solution $\hat{z}_0$.

While $\sum_{i=1}^{N}(|F_i(D\hat{z}_k)|^2 - y_i)^2 > \tau$ or $k \leq MaxIter$ ***do***

$k = k + 1$

Construct new dictionary D based on solution $\hat{z}_{k-1}$. For each non zero index of $\hat{z}_{k-1}$, for example α_i, construct 9 bars in D as follows: 3 bars with widths $w + \delta$, $w - \delta$ and w positioned in α_i, 3 bars with same widths positioned in $\alpha_i - 1$ and 3 bars with the same widths positioned in $\alpha_i + 1$.

Solve problem (1) with new dictionary and obtain solution $\hat{z}_k$.

End While

Return $\hat{z} = \hat{z}_k$

Supplementary Note 4

Sparsity-based super-resolved 1D CDI of objects that are sparse in a frame of rectangles with various widths.

The “sparsity basis” is an important component in sparsity-based CDI (in all dimensions). The basis that was used in the paper - rectangle functions with a fixed width – is not an essential component of the method. Below we demonstrate an extension of this basis into a frame of rectangular functions with various widths. The sparsity frame in the example consists of shifted rectangles with widths that vary between 5 um and 30 um in steps of 5um. Supplementary Figure 4 shows an example for a super-resolved 1D CDI of a localized piecewise constant object. Supplementary Figure 4 a shows a signal (which we term the “original object”) consisting of 7 rectangles with various widths, amplitudes and centers (some rectangles overlap). The power spectrum of the original object is shown in Supplementary Figure 4 b. To demonstrate super-resolution, we truncate the power spectrum and add 43dB of white Gaussian noise to obtain the “truncated power spectrum” emulating a physical measurement in a typical CDI system (Supplementary Figure 4 c). Reconstruction requires retrieval of the spatial spectral phase. Figure S.4d displays the object that corresponds to the truncated power spectrum of Supplementary Figure 4 c, while assuming that the correct spectral phase was retrieved (in this example, the correct spectral phase is simply calculated by a Fourier transform of the original object (Supplementary Figure 4 a)). Naturally, this reconstructed object (Supplementary Figure 4 d) is a blurred version of the original object. That is, the incomplete power spectrum has led to considerable loss of resolution even if the spectral phase is known. Next, we implement sparsity-based reconstruction on the truncated spatial power spectrum, without assuming any knowledge on the spectral phase. As a model, we assume that the object is constructed from a small

(unknown) number components in our frame. The sparsity-based GESPAR reconstruction algorithm finds the number of rectangles, their locations, their widths and their amplitudes from the truncated power spectrum (Supplementary Figure 4 c). The reconstructed object is shown in Supplementary Figure 4 e. Its power spectrum and spectral phase are displayed in Supplementary Figure 4 f and g, respectively. To enable comparison, Supplementary Figure 4 e-f also show the original object. Clearly, the reconstructed object, its complete power spectrum and its reconstructed spectral phase match the original object very well despite usage of the noisy truncated spectrum as “measured data” and the lack of any knowledge on the spectral phase.

Supplementary References

1. Shechtman, Y., Beck, A. & Eldar, Y.C. GESPAR: Efficient Phase Retrieval of Sparse Signals. *IEEE Transactions on Signal Processing* **62**, 928-938 (2014).
2. Bruck, Y.M. & Sodin, L.G. On the ambiguity of the image reconstruction problem. *Optics Communications* **30**, 304-308 (1979).
3. Taylor, L. The phase retrieval problem. *IEEE Transactions on Antennas and Propagation* **29**, 386-391 (1981).
